# Author Correction: Modeling COVID-19 scenarios for the United States

**DOI:** 10.1038/s41591-020-01181-w

**Published:** 2020-11-27

**Authors:** IHME COVID-19 Forecasting Team, Robert C. Reiner, Ryan M. Barber, James K. Collins, Peng Zheng, Christopher Adolph, James Albright, Catherine M. Antony, Aleksandr Y. Aravkin, Steven D. Bachmeier, Bree Bang-Jensen, Marlena S. Bannick, Sabina Bloom, Austin Carter, Emma Castro, Kate Causey, Suman Chakrabarti, Fiona J. Charlson, Rebecca M. Cogen, Emily Combs, Xiaochen Dai, William James Dangel, Lucas Earl, Samuel B. Ewald, Maha Ezalarab, Alize J. Ferrari, Abraham Flaxman, Joseph Jon Frostad, Nancy Fullman, Emmanuela Gakidou, John Gallagher, Scott D. Glenn, Erik A. Goosmann, Jiawei He, Nathaniel J. Henry, Erin N. Hulland, Benjamin Hurst, Casey Johanns, Parkes J. Kendrick, Apurva Khemani, Samantha Leigh Larson, Alice Lazzar-Atwood, Kate E. LeGrand, Haley Lescinsky, Akiaja Lindstrom, Emily Linebarger, Rafael Lozano, Rui Ma, Johan Månsson, Beatrice Magistro, Ana M. Mantilla Herrera, Laurie B. Marczak, Molly K. Miller-Petrie, Ali H. Mokdad, Julia Deryn Morgan, Paulami Naik, Christopher M. Odell, James K. O’Halloran, Aaron E. Osgood-Zimmerman, Samuel M. Ostroff, Maja Pasovic, Louise Penberthy, Geoffrey Phipps, David M. Pigott, Ian Pollock, Rebecca E. Ramshaw, Sofia Boston Redford, Grace Reinke, Sam Rolfe, Damian Francesco Santomauro, John R. Shackleton, David H. Shaw, Brittney S. Sheena, Aleksei Sholokhov, Reed J. D. Sorensen, Gianna Sparks, Emma Elizabeth Spurlock, Michelle L. Subart, Ruri Syailendrawati, Anna E. Torre, Christopher E. Troeger, Theo Vos, Alexandrea Watson, Stefanie Watson, Kirsten E. Wiens, Lauren Woyczynski, Liming Xu, Jize Zhang, Simon I. Hay, Stephen S. Lim, Christopher J. L. Murray

**Affiliations:** 1 Institute for Health Metrics and Evaluation, University of Washington, Seattle, WA USA; 2 Department of Health Metrics Sciences, School of Medicine, University of Washington, Seattle, WA USA; 3 Department of Political Science, University of Washington, Seattle, WA USA; 4 Center for Statistics and the Social Sciences, University of Washington, Seattle, WA USA; 5 Department of Applied Mathematics, University of Washington, Seattle, WA USA; 6 Queensland Centre for Mental Health Research, The University of Queensland, Brisbane, Queensland Australia; 7 School of Public Health, The University of Queensland, Brisbane, Queensland Australia; 8 Queensland Centre for Mental Health Research, Brisbane, Queensland Australia; 9 Big Data Institute, University of Oxford, Oxford, UK; 10 Department of Global Health, University of Washington, Seattle, WA USA; 11 QIMR Berghofer Medical Research Institute, Brisbane, Queensland Australia; 12 Henry M Jackson School of International Studies, University of Washington, Seattle, WA USA; 13 Policy and Epidemiology Group, Queensland Centre for Mental Health Research, Brisbane, Queensland Australia

**Keywords:** Infectious diseases, Health policy

Correction to: *Nature Medicine* 10.1038/s41591-020-1132-9, published online 23 October 2020.

In the Supplementary Information (item 4.4.1, Data processing) of the version of this article initially published, the date range of data incorporated in the reported analyses (23 April 2020 to 26 June 2020) was incorrect. The correct date range is 23 April 2020 to 21 September 2020, as indicated in the main text of the manuscript. The paper and its conclusions are unaffected by this correction to the Supplementary Information. The Supplementary Information has been replaced online with an updated version.

